# Materia Medica and Therapeutics

**Published:** 1861-11

**Authors:** Emil Fischer


### MATERIA MEDICA AND THERAPEUTICS; MEDICAL CHEMISTRY; TOXICOLOGY AND LEGAL MEDICINE.

BY EMIL FISCHER, M.D.

### MATERIA MEDICA AND THERAPEUTICS.

### I.—*Solution of Phenic Acid in Tinea and Scabies.* By M. BAZIN.

According to the experiments made at the hospital *Saint-Louis*, in the service of M. Bazin, and communicated by M. Jean Lemaire to the *Académie des Sciences*, the following solution constitutes a very economic remedy of scabies and tinea:—

℞.—Phenic acid, 1 part;
Acetic acid at 8 degrees, 40 parts;
Water, 100 parts.

In the treatment of tinea a compress soaked in this solution is applied once a day. In scabies, a single lotion is sufficient to kill the ascarus. The acetic acid is added to the preparation in order to make the remedy penetrate beneath the epidermis and to the bottom of the hair bulbs.

Phenic, or carbolic acid, is a product obtained by the distillation of coal, and has, consequently, but little venal value; the author has likewise reflected upon the hygienic applications this acid could receive. The most important is the preservation of dead bodies. M. Lemaire states that the cadaver of a man could be maintained in an excellent state of preservation for less than fifty centimes.—(*Bulletin Général de Thérapeutique*, and *Journ. de Pharnacie*, July, 1861.)

### II.—*On the Use of Cinchonine in the Treatment of Intermittent Fever.*

In the General Hospital of Munich cinchonine has been for several years employed very successfully in the treatment of intermittent fever. The quantity administered during a day is fifteen grains, divided into two doses, of which the first contains ten, and the second five grains. It is necessary, for a complete cure, to give this quantity three or four times, at intervals of three to four days. From the favorable results obtained in the hospital mentioned above, no definite conclusion can, however, be drawn, as intermittent fever in Munich is generally an imported disease, and not as obstinate as in districts where it is endemic. In order to test the efficacy of cinchonine on a greater scale, and with a view of substituting this article for quinine on account of its greater cheapness, the Bavarian government has ordered experiments to be made in the prisons and other public institutions under its control.—(*Neues Repertorium für Pharmacie*, X, 4 and 5, 1861.)

### III.—*Iodine in Tubercular Meningitis.* By DR. BOURROUSSE.

Dr. Bourrousse, at the conclusion of a long memoir laid before the Académie de Médecine, states: 1. All, or almost all, cases of tubercular meningitis, which have resisted hitherto all therapeutical procedures, may now be deemed curable. 2. One or two hundred thousand infants will owe their lives each year to the employment of iodide of potassium in acute hydrocephalus. 3. This meningeal affection is the only tubercular disease which has been rendered curable, and this success will probably lead to amendment in the mode of treating other forms of tuberculization.—(*Moniteur des Sciences Med.*, No. 73, 1861.)

IV.—*Combination of the Essential Oil of Matico with Balsam of Copaiba.* By Dr. Favrot.

Matico (*piper angustifolium*) is a pepper-shrub of South America, introduced into France by M. Dorvault, who brought it from the World's Fair of London. The principal property of matico is that of an astringent.

At the same period some English physicians conceived the idea of administering the decoction internally in cases of chronic discharges from the uretha. In Peru, the remedy had been employed for a long time with the same intention. In the *Flore Médicale* of that country, page 28, the following passage can be read: "*Incolæ ad ulceras cancrosas et gonorrheas a lue venerea ortas decoctum affatim hauriunt.*"

After M. Dorvault, his successor, M. Grimaud, continued the experiments, and arrived at the conclusion that the properties of matico are owing to the essential oil which this plant contains in large quantity. M. Favrot prepared capsules with this oil, administered them, and obtained cures in the majority of cases where he employed them.

The author found that the diuretic action was too intense, and, in order to modify it, had the idea of combining the oil of matico with balsam of copaiba. The following is the formula of this preparation:—

| | |
|---|---|
| Balsam of copaiba, | 100; |
| Essential oil of matico, | 5; |
| Magnesia, | q. s. |

For one hundred boles, to be covered with gluten according to the method of M. Raquin. Each bolus contains one gramme of copaiba and fifty centigrammes of essential oil of matico, which represents an equivalent of fifty grammes of leaves employed in extracting it.

In this combination the balsam loses its nauseous taste as much as with peppermint. The effect of these boles in the mentioned cases was remarkable: "It is nearly four years," says M. Favrot, "that I treat with this preparation acute and chronic discharges with advantage, if I compare it with the analogous preparations which have been employed until the present day."

The local treatment is conducted by injections with distilled water saturated with matico, which M. Grimaud prepares in the following manner:—

Matico, 1 kilogr;
Water, 2 "

To obtain 1000 of saturated water, he macerates the matico in cold water for twenty-four hours before distillation. For the general or internal treatment the boles or capsules, of which the formula has been given above, must be used.—(*Journal de Pharmacie et de Chimie*, July, 1861.)

V.—*Electuarium of Cubeba, Copaiba, and Matico.* By M. DEBOUT.

The most efficacious, and at present most employed, preparations in the treatment of blennorrhagia being electuaries resulting from the combination of cubeba and copaiba, M. Debout does not hesitate in recommending the following formula for experiment:—

| | |
|---|---|
| Copaiba, | 30 grammes; |
| Cubeba, | 45 " |
| Essence of matico, | 2 " |
| Powdered sugar, | q. s. |

To be taken in three days, enveloped in unleavened bread. Each daily quantity should be divided into at least four doses, so as to maintain the urine constantly in a medicated state.—(*Bulletin de Thérapeutique*, and *Jour. de Pharmacie*, July, 1861.)

VI.—*Distilled Water of Copaiba.* By DR. EDWARD LANGLEBERT.

Dr. Langlebert calls attention to this new distilled water, which he has employed about a year in urethral blennorrhagia.

Every physician knows that copaiba taken in the stomach acts locally after passing the kidneys, by aid of the urine, and this action has been attributed to a certain modification which it undergoes by the *vital chemistry* of the economy. The author views it as a case of distillation, in which the urine becomes the aqueous solvent of the oil of copaiba.

It is this idea which suggested to him the employment of the Distilled Water of Copaiba as a vehicle for urethral injections. The following are some of the injections which he has most frequently prescribed:—

Distilled water of copaiba, three fluid ounces;
Sulphate of zinc, six grains;
Tincture of catechu, fifteen minims.

Mix.

Distilled water of copaiba, three fluid ounces;
Sulphate of zinc, four and a half grains;
Lapis divinus, one and a half grains.

Mix.

Distilled water of copaiba, three fluid ounces;
Sulphate of zinc, six grains;
Levigated oxide of zinc, a drachm.

Mix.

Distilled water of copaiba, three fluid ounces;
Tannic acid, or catechu, fifteen grains.

Mix.

Numerous experiments have convinced the author that these injections are superior to similar preparations in which distilled water or rose water are used in lieu of the copaiba water.

Copaiba water is prepared by simply distilling water along with copaiba, and separating the supernatent volatile oil. A metallic still is to be preferred, owing to the bumping that occurs in a glass retort.

Administered internally, its effects are like those of copaiba, but milder. It has been prescribed in quantities of four to six ounces per diem, with a few drops of cherry-laurel water, to cover its taste. It is taken with less repugnance, and has never caused pain in the kidneys, frequently produced by ordinary copaiba.—(*Gazette des Hôpitaux*, and *Am. Journal of Pharmacy*, September, 1861.)

### VII.—*Formula for the Administration of Chloroform, Ether, Turpentine, Camphor, or Essential Oils.*

M. Vee recommends the following as a valuable means for the equable administration of these medicinal substances: Chloroform, (or any of the above,) 4 parts, (or less;) oil of sweet almonds, 15 parts; powdered gum arabic, 10 parts; water, 100 parts; syrup, 25 parts. The chloroform is to be dissolved in the oil, and rapidly emulsified, to prevent evaporation. *Syrup of Chloroform* may be well prepared by the following formula: 10 parts by weight of chloroform are to be dissolved in 60 of oil of sweet almonds, 40 parts of gum added, and an emulsion formed with 350 parts of water. In this cold emulsion, placed in a close vessel, 540 parts of sugar are to be dissolved. It is a very stable syrup, rendering water white on admixture with it, and it contains exactly one-hundredth of its weight of chloroform.—(*Med. Times and Gaz.*, June 8, 1861, from *L'Union Méd.*, No. 49.)

### VIII.—*Ferri Carbonas Effervescens: a New Form of Chalybeate.*

Dr. T. Skinner, in a communication to the Dublin Medical Press, recommends the following formula for preparing effervescing carbonate of iron:—

| | |
|---|---|
| ℞.—Acidi Tart. | ℥iij; |
| Sodæ Bicarbonatis, | ℥v; |
| Ferri Sulph. | ʒx; |
| Pulv. Sacchari, | ℥j, ʒvj; |
| Acidi Citrici, | ʒij. |

1. Mix the sulphate of iron with the sugar and part of the tartaric acid. 2. Mix the citric acid with the remainder of the tartaric acid and the bicarbonate of soda. 3. Add the mixtures, and thoroughly incorporate them by sifting. 4. The whole is now to be thrown into a metallic pan set in a water bath; in a few minutes it will separate, when it should be rapidly stirred until granules are formed. If preferred, it may then be flavored with oil of lemon; hitherto, however, the preparation has been without it.

When the above is carefully prepared, it has all the appearance of the popular and well known granular effervescent citrate of magnesia, with the addition of a slight yellowish-green tint. Every drachm and a half contains ten grains of sulphate of iron, which, with a complement of bicarbonate of soda, is certain to produce, in a state of solution, four grains of nascent protocarbonate of iron.—(*London Pharm. Journ.*, August, 1861.)

IX.—*Abortion in Cows occasioned by the Ingestion of Ustilago Madis.*

This ustilago is a parasitic mushroom, which occurs on maize as ergot does on rye. In a cow-house, where cows were fed on Indian-corn infected with this parasite, eleven of their number aborted in eight days. After their food was changed, none of the others aborted. The better to be convinced of the poisonous nature of these mushrooms, the author, after having dried and pulverized them, administered six drachms to two bitch-dogs with young, which soon caused them to abort.—(*Annal. Méd. vetr. Belge*, and *Am. Journ. of Pharmacy*, September, 1861.)

X.—*Alcohol—Its Action and Uses.*

The question of the action of alcohol on the system, and its value as a dietetic and therapeutic agent, is now attracting much attention, both among the members of our profession and the scientific world at large. No doubt can be entertained of the importance of a consideration of the subject—a consideration, however, into which our limits will not allow us fully to enter. If there were one cherished view which we thought chemistry had taught us, it was surely that alcohol was oxidized in the system, and thus made subservient, if not to the formation of some of the tissues of the body, at any rate to the maintenance of animal heat. However indisposed physiologists may have been of late years to give in their adhesion to the theories of Liebig as to the division of alimentary materials, we think that most have been, and probably many will still be, disposed to consider the substance we are speaking of in the light of a heat-producing agent. Our opinions on this point have, however, been somewhat rudely assailed, and the position which alcohol has occupied as a dietetic article is threatened with imminent danger. As from the chemists we received the theory of its combustible nature, so from chemical research and experiment now comes the opposite doctrine of its entire elimination, unchanged, from the body.

Considering the vast amount of alcohol which is daily consumed, whether as a general article of diet, or in the treatment of disease, it becomes a matter of essential importance that correct principles should be laid down with reference to its *modus operandi*, and the manner in which it is disposed of in the system. We cordially hail, therefore, the appearance of the work of Messrs. Lallemand, Perrin, and Duroy, (*Du Rôle de l'Alcohol et des Anesthésiques dans l'Organisme*, Paris, 1860,) which gives us the results of the most recent experiments and researches on the subject. A careful perusal of this work has convinced us that some of our views of the dietetic value of alcohol require modification; but we cannot, on that account, allow the evidence which is brought forward, and which does not appear to us conclusive on the point, to make us discard altogether the substance of which we are speaking from our list of alimentary materials.

If we were briefly to sum up the facts which the researches of the above authors seem to have established, they would be these: That, after any fluid containing alcohol is taken, the latter becomes eliminated, unchanged, by the various secreting organs—the skin, the liver, the kidneys, the lungs; and not only this, but that it is deposited in all the tissues, and can be extracted alike from the substance of the brain, the liver, the muscles, and the cellular tissue, as well as from the blood. It is right to observe that, even when small quantities of alcohol were taken, traces of it could be found in the excretions; thus proving that it is not simply when the substance is taken in excess that it becomes eliminated without change.

From the results of their experiments, MM. Lallemand, Perrin, and Duroy think they are justified in concluding that all the alcohol ingested, with the exception of a small quantity which they and other experimenters have found converted into acetic acid in the stomach, is eliminated from the body, without undergoing any change whatever; and that in this respect it resembles in its action the various anæsthetic agents, such as chloroform, etc. From these conclusions necessarily follows the inference that alcohol cannot be considered in the light of an alimentary substance.

We believe Dr. Percy was the first to show that, after poisoning by alcohol, this substance could be found in the ventricles of the brain, as well as in the brain-matter itself; and we are all aware of the fact that fumes of alcohol are exhaled from the lungs even when only a small quantity of the fluid has been swallowed. The chief points that are new, which have been brought to light by the investigations of our authors, are the elimination of the alcohol, unchanged, through the medium of the skin, the kidneys, etc.; and the deposit in the tissues generally. It is true that Klenke had demonstrated the presence of alcohol in the urine and bile, but, with this exception, physiologists had advanced the opinion that the secreting organs did not eliminate the substance.

There cannot be a doubt of the great importance and value of the researches of M. Lallemand and his coadjutors; for supposing their results to be trustworthy—and we believe them to be so—they establish beyond a doubt the fact that, at any rate, a portion of all ingested alcohol may go the round of the circulation, traverse the various capillaries of the body, and, without undergoing any oxidation whatever, be ejected from the system in the different secretions. This portion, therefore, cannot be concerned in building up the fabric of the body, nor can it by its combustion contribute to the maintenance of animal heat. This is surely an important conclusion to have arrived at. The weak point, however, in the arguments adduced against the value of alcohol as a respiratory food, is the small quantity of it which the experimenters have been able to extract from the various secretions. It is possible that, by more delicate modes of investigation, and by the use of more subtile tests, a much larger proportion may be detected; but we are inclined to think that, until some such result is obtained, physiologists will not be disposed to abandon their views as to the oxidizable nature of the substance.—(*London Pharm. Journ.*, May, 1861, from *Br. Med. Journ.*)

### TOXICOLOGY AND LEGAL MEDICINE.

### I.—*Duty of a Physician to the Public in a Case of Poisoning.*

M. Tardieu, treating of this important question in medical deontology, in his lectures on medical jurisprudence at the faculty, communicated to his auditors a letter written by Lammenais to Dr. Pierquin, of Montpellier. "You have done me the honor of asking my opinion on the question, 'Is a practitioner, who perceives that a patient to whom he has been called has been poisoned, under the moral obligation of declaring his well-founded opinion to the authorities?' Without doubt, in many cases, a mere private person is not obliged to reveal to the authorities a crime of the certain existence of which he is aware, and sometimes charity may even render it a duty on his part to observe silence. But is this the case with the medical practitioner? On the contrary, is he not in an essentially different position? The physician is a public man, and he has special duties toward society which ensue from his very function, and he should advertise it of the crimes which he alone is in a condition to discover and bring home; otherwise these crimes, which are always among the most heinous, would never be known, except by some extraordinary contingency, especially now, when the art of poisoning has made such fearful progress, and when crime seems to have taken refuge in the bosoms of families, destroying all security of life. The confessor is compelled for other reasons. It is almost always the criminal who comes to him, and his conscience is a sanctuary he cannot leave. But the physician who discovers that which, so far from being confessed to him, is sought to be hidden from him, has two duties to perform—one toward his patient, and the other toward society, whose minister he is on this occasion; and if, as cannot be doubted, he ought to advertise the magistrate, when a disease exhibits alarming signs of contagion, how much more is he called upon to reveal that which implicates not some person only, but society itself?" The *Union Médicale*, while adhering to the above doctrine, cautions its readers that excessive care and circumspection should be employed before making any spontaneous declaration of the kind. Mere doubts are not to be revealed, these requiring first to be converted into a settled conviction and a commencement of certainty. —(*Med. Times and Gaz.*, July 6th, 1861.)

### II.—*What constitutes Evidence of Live Birth.*

Vice-Chancellor Sir J. Stuart decided a very curious point in medical jurisprudence, the other day, in the trial of Brock *v.* Kellock. Upwards of twenty years ago, a medical man died without a will, leaving property and a widow, who was pregnant at the time of his death. As his widow, she succeeded to a portion of the effects; but in case of her having a child, and the child dying, she became, as the heir of her own offspring, entitled to a further portion. She was delivered by Mr. Freeman, now of Plymouth; and the question was, did the mother give birth to a live or dead child? Mr. Freeman distinctly stated, in his affidavit, that the funis pulsated after the separation of the cord, and that the child moved vigorously a minute before birth. He could not swear to the fact of respiration having taken place, but he remembered that the chest was full and arched, and he believed it must have breathed, because it was his usual practice not to divide the funis until respiration had taken place. He also placed the child in a warm bath, and he stated that he should not have done this, had he not considered the child alive and capable of resuscitation. His distinct opinion was, that the child was born alive, but weak; and that it died shortly after birth from the effects of a severe labor.

Dr. Robert Lee, in an affidavit, contested, in the strongest manner, the statements and opinions of Mr. Freeman. He thought it very likely that the pulsation in the finger and thumb might have been mistaken for the funic pulse. He maintained that the action of the heart, supposing it to have taken place, was no proof of life, such as should be established, because the heart of animals would contract for hours or days after separation from the body. In his opinion, respiration was necessary to establish the fact of live birth. He further said, Mr. Freeman had testified that respiration had not taken place, and that no medical man would dare to give a certificate of live birth in such a case. Dr. F. H. Ramsbotham concurred generally in the affidavit of Dr. Lee.

Dr. Tyler Smith, in his affidavit, said that the pulse of the adult and the beat of the funis were so different, that the mistake supposed by Dr. Lee was not likely to have been made. He contended that a child, whose heart was beating after its separation from the mother, could not be said to be dead; that the beating of the heart of the lower animals, after separation from the body, could not apply to the human infant; and that even in these cases, it was an evidence of vitality.

There was no proof of respiration having taken place; but Mr. Freeman's opinion upon this point was the best that could be had, and Dr. Smith showed that Dr. Lee was wrong in stating that Mr. Freeman had said no respiration occurred. He thought medical evidence was limited to the proof of life or death in a physiological point of view; that the law must decide what constituted legal life. He mentioned that medical men sometimes gave certificates of still-birth when the child moved and made attempts at respiration, if it did not fully recover itself after birth. His conclusion was, that it would be better to take a single vital act, such as the beating of the heart, which had been considered by physiologists the *primum vivens, ultimum moriens*, as a proof of live birth, than the movements of respiration, which were often not fully established for some time after birth.

The Vice-Chancellor, in his dicision, entirely affirmed Dr. Tyler Smith's view of the case, the good sense of which he greatly commended, and expressed his surprise that a man in Dr. Lee's position should have made such an affidavit. He held it proved that the child had been born alive, and refused the costs of the medical evidence on the opposing side.

Dr. Alfred Taylor made no affidavit in the case, but was appealed to by Dr. Lee and gave an opinion in favor of live birth. This decision is of importance, inasmuch as it will go far to form the law, which has hitherto, in this country, been unsettled upon the point at issue.—(*Lancet*, May 11th, 1861.)

